# Synthesis and Engineering of Hyaluronic Acid-Gelatin Hydrogels with Improved Cellular Attachment and Growth

**DOI:** 10.3390/polym16233410

**Published:** 2024-12-03

**Authors:** Alma Tamunonengiofori Banigo, Irene B. M. Konings, Laura Nauta, Bram Zoetebier, Marcel Karperien

**Affiliations:** Department of Developmental BioEngineering, Faculty of Science and Technology and TechMed Centre, University of Twente, Drienerlolaan 5, 7522 NB Enschede, The Netherlands; a.tamunonengioforibanigo2025@outlook.com (A.T.B.); i.b.m.konings@utwente.nl (I.B.M.K.); l.nauta@utwente.nl (L.N.); b.zoetebier@utwente.nl (B.Z.)

**Keywords:** hyaluronic acid hydrogels, gelatin, cell morphology, cartilage matrix formation

## Abstract

Injectable hydrogels are promising materials for cartilage regeneration in tissue engineering due to their tunable crosslinking rates, mechanical properties, and biodegradation profiles. This study investigates the chondrogenic potential of hyaluronic acid (HA) hydrogels crosslinked via tyramine (TA) moieties, with and without gelatin modified with TA (Gel-TA). Incorporating Gel-TA improved cell viability, spreading, and cartilage matrix deposition, particularly in medium and high molecular weight (MMW and HMW) HA-TA/Gel-TA hydrogels. Although the hydrogels’ molecular weight did not significantly alter stiffness, MMW and HMW HA-TA/Gel-TA formulations exhibited enhanced functional properties such as slower degradation and superior cartilage matrix deposition. These attributes, coupled with Gel-TA’s effects, underscore the importance of both molecular weight and biofunctional components in hydrogel design for cartilage regeneration. While low molecular weight (LMW) HA-TA hydrogels offered excellent injectability and supported high cell viability, they degraded rapidly and exhibited reduced cartilage matrix formation. Gel-TA enhanced cell adhesion and spreading by providing integrin-binding sites and promoted collagen type II deposition, crucial for cartilage regeneration. Moreover, the increased stiffness of MMW and HMW HA-TA/Gel-TA hydrogels facilitated extracellular matrix production. These findings show the potential of Gel-TA-modified HA-TA hydrogels for cartilage tissue engineering, with the opportunity for further optimization through the incorporation of bioactive components.

## 1. Introduction

The ideal hydrogels (3D scaffolds) for tissue regeneration should exhibit excellent chemical properties (such as controlled degradation and crosslinking), physical properties (including optimal biomechanics, diffusion, and porosity), and biological properties (such as bioactive cues, compatibility with specific cell types, and the ability to incorporate growth factors) [1,2]. They can be considered for 3D bioprinting with high shape fidelity and stiffness. In addition to their tuneable mechanical properties, they require good tuneable biological properties. While some cells perform in non-adhesive hydrogels, others can only thrive with adhesive factors [3].

Hyaluronic acid (HA) is a natural linear anionic polysaccharide that is present in the extracellular matrix (ECM). One of the crucial features of HA is its molecular weight (MW) [4], determining the biological effects, degradability, structural, physical, and physicochemical properties [5], hydrodynamic size [5], and physiological activity [5,6,7]. Understanding the effect of the MW of HA related to articular cartilage (AC) regeneration gives insight into the interactions of HA within the joint and HA’s influence on AC repair processes. HA can be roughly divided into three MWs: low (LMW; <250 kDa), medium (MMW; 250–1000 kDa), and high (HMW; >1000 kDa) [8]. Some properties of LMW, MMW, and HMW HA are briefly outlined in Supplementary Materials: Table S1. Despite the clear difference in physicochemical and biological properties of different molecular weights of HA, a detailed side-by-side comparison of how these differences can influence the properties and corresponding effects of hydrogels in tissue engineering are lacking. 

LMW HA is excellent for injectability [9,10] due to its low viscosity, high cell viability, and other properties, and has been used for 3D bioprinting [11,12,13,14], as well. MMW and HMW HAs are moderately and highly viscous, respectively, with unique properties that can be applied to the 3D bioprinting of tissue [15,16].

Previous studies performed by our group have shown the injectability of LMW HA (25 kDa) and Dextran [Dex] (40 kDa) to produce hybrid hydrogels [17,18,19,20] with good mechanical and biological properties. Next to that, we also explored the use of enzymatically crosslinked HMW HA-TA (2.0–2.2 MDa) for the coaxial bioprinting of core filaments. Our results showed good cell viability above 80% on day 0 for cell-laden core filaments but decreased viability over time due to a lack of some biological properties. The addition of gelatin-tyramine (Gel-TA) to polysaccharide hydrogels could be an outcome of this, as we have shown that this improves their biological properties [3].

The objective of this study was twofold: We performed a side-by-side comparison of the chondrogenic potential of hydrogels made of LMW, MMW, and HMW HA-TA and evaluated the potential beneficial effects of adding low concentrations of Gel-TA to these hydrogels. We compared the mechanical properties, swelling ratio, degradation ratio, viability of cells, and formation of cartilage matrix in all the hydrogels with different MWs and Gel-TA additions.

## 2. Materials and Methods

### 2.1. Materials

Low, medium, and high molecular weights (LMW, MMW, and HMW) of hyaluronic acid (HA) sodium salt from Streptococcus equi with a weight-average molecular weight of 27 kDa, 130–300 kDa, and 2.0–2.2 MDa, respectively, were purchased from Contipro Pharma, Dolní Dobrouč, Czech Republic. Gelatin from porcine skin (300 g bloom type A) was purchased from Sigma, St. Louis, MO, USA, and 4-(4,6-dimethoxy-1,3,5-triazin-2-yl)-4-methylmorpholinium chloride (DMTMM, 97%) was obtained from Fluorochem Ltd., Hadfield, UK. Tyramine hydrochloride (TA∙HCL, 99%), deuterium oxide (D_2_O, 99.9 atom % D), horseradish peroxidase (HRP, 250 U/mg), hydrogen peroxide (H_2_O_2,_ 30%), and other reagents were purchased from Sigma-Aldrich, Schnelldorf, Germany. Ethanol (≥99.9%) and diethyl ether (≥99.7%) were purchased from Merck, Kenilworth, NJ, USA. Milli-Q water was used from the Milli-Q Advantage A10 system equipped with a 0.22 μm Millipak^®^-40 Express filter (Merck KGaA, Darmstadt, Germany). Chemicals were used without further purification.

### 2.2. Methods

#### 2.2.1. Synthesis of LMW, MMW, and HMW Hyaluronic Acid-Tyramine

Hyaluronic acid-tyramine conjugates were synthesized by a one-step amidation of the HA carboxyl groups with tyramine, as reported by Rydergren and D’Este et al. [21,22]. The detailed polymer synthesis can be found in Supplementary Materials: Protocol No. 1a and b.

We prepared LMW and MMW HA-TA with a substitution degree (DS) of 10% and HMW HA-TA with a DS of 6.5%. The DS of the hyaluronic acids is given as the percentage of carboxyl groups modified in hyaluronic acid (i.e., per 100 disaccharide units).

#### 2.2.2. Synthesis of Gelatin-Tyramine

Gelatin (5.00 g) from porcine skin (300 g bloom type A, Sigma, USA) was dissolved in 500 mL Milli-Q water in a round-bottomed flask equipped with a PTFE magnetic stirrer bar at 40 °C until completely dissolved. Once cooled down to 35 °C, the gelatin was chemically modified and purified as described for HA-TA. The product obtained was a white foam (3.9 g, 78% yield, DS 16.5%).

The degree of substitution (DS) was calculated based on the increase in absorption at 275 nm in the UV–visible spectra of gelatin-TA (Gel-TA) and a tyramine calibration curve. The DS of the gelatin is given as the percentage of carboxylic amino acids modified in gelatin [3].

The chemical structure and hydrogel formation of HA-TAs of different molecular weight and Gel-TA are depicted in Supplementary Materials: Figure S1.

#### 2.2.3. Hydrogel Formation

Cylindrical hydrogel samples were prepared in a PTFE mold (Supplementary Materials: Figure S2) to form identical cylindrical (8 mm diameter and 1.5 mm height) hydrogels as described by Fu et al. [9]. Tyramine-modified polymers were dissolved in phosphate-buffered saline (PBS) and incubated in the presence of HRP overnight at 4 °C. Six conditions were studied: LMW HA-TA, LMW HA-TA/Gel-TA, MMW HA-TA, MMW HA-TA/Gel-TA, HMW HA-TA, and HMW HA-TA/Gel-TA at 2.5% *w*/*v* HA-TA and 0.05% *w*/*v* Gel-TA polymer concentrations.

Here, 0.25 and 1.0 U/mL HRP were used to produce LMW HA-TA and LMW HA-TA/Gel-TA hydrogels whereas 0.25 U/mL HRP only was used to produce MMW HA-TA, MMW HA-TA/Gel-TA, HMW HA-TA, and HMW HA-TA/Gel-TA hydrogels.

Bovine primary chondrocytes were added to yield a concentration of 10 million cells/mL.

For hydrogel preparation, freshly prepared 0.0474% hydrogen peroxide (H_2_O_2_) at a 1:1 TA:H_2_O_2_ molar ratio (adapted for HMW HA-TA to 1:1.5 to keep the same number of crosslinks in all conditions) was added to the mixture, stirred with a magnetic stirrer, and transferred into a PTFE mold with a 1 mL positive displacement pipette immediately after mixing.

#### 2.2.4. Rheological Properties

The viscoelastic properties of the cylindrical hydrogel samples were measured using HR 20 Discovery Hybrid Rheometer-TA Instruments (New Castle, DE, USA) equipped with a Peltier stage temperature control system and parallel plates (ø 8 mm) geometry.

Measurements were taken at 20 °C (+/−0.2 °C) under an initial normal force of 0.05 N, within the linear viscoelastic (LVE) range, at 0.5% strain and 1.0 Hz frequency. The hydrogel samples were equilibrated for 24 h in 1 mL of PBS at 4 °C before measurement.

A minimum of three hydrogel samples were measured for each of the six conditions. 

#### 2.2.5. Compression Test

Compression testing was performed on the hydrogel samples (described in “Hydrogel formation”) with the use of HR 20 Discovery Hybrid Rheometer-TA Instruments (New Castle, DE, USA). The hydrogels underwent a single compression cycle with a maximum strain of 30% at a compression speed of 0.05 mm/s. The compression tests were conducted at room temperature and at least three specimens were tested for each formulation. 

#### 2.2.6. Swelling Ratio Measurement

The swelling ratio was assessed by measuring the weight of each hydrogel after overnight equilibration in PBS compared to its dry weight. At least three samples were measured for each condition. The swelling ratio calculation was based on the weight of each hydrogel where the water content ($w_{w}-w_{d}$) was divided by the polymer content (dry weight, $w_{d}$), as shown in Equation (1):

(1) $$\mathrm{Swelling}\;\mathrm{ratio}=\frac{w_{w}-w_{d}}{w_{d}}.$$

#### 2.2.7. Enzymatic Degradation

All prepared hydrogel samples were incubated in 1 mL of PBS at 4 °C for 24 h and weighed to determine their initial weight (w_i_). For the hydrogel degradation, we incubated the hydrogel samples in 1 mL of either 2.5 U/mL or 5 U/mL hyaluronidase in PBS. The change in weight of the hydrogel samples was measured during incubation at 37 °C for 5 days (w_c_). The percentage of the remaining hydrogel samples was determined using the Equation (2) below [23]:

(2) $$\mathrm{Degradation}\;\mathrm{ratio}\;\left(\%\right)=\frac{w_{c}}{w_{i}}\times 100\%.$$

#### 2.2.8. Cell Culture and Expansion

Bovine chondrocytes (bCHs) were isolated from cartilage knee biopsies of full thickness from calves and cryopreserved. The bovine primary chondrocytes (BPCs) were thawed and cultured in a chondrocyte proliferation medium (Dulbecco’s Modified Eagle Medium (DMEM; Gibco, Billings, MT, USA) and supplemented with 10% fetal bovine serum (FBS; Sigma S0615, Lot no. 0001652821), 0.2 mM of ascorbic acid 2-phosphate (Sigma), 0.4 mM of proline (Sigma), 1× nonessential amino acids (Gibco, New York, NY, USA), 100 U/mL of penicillin, and 100 μg/mL of streptomycin (Invitrogen, Carlsbad, CA, USA). The medium was refreshed twice per week and 10 million cells per mL were used for experiments at 80% confluency and in passage 3.

#### 2.2.9. Live/Dead Cell Viability Assay

The cell-laden hydrogels were evaluated using a live/dead cell viability assay (Invitrogen), according to the manufacturer’s instructions, on days 1, 7, and 13. All samples were incubated at 37 °C and 5% CO_2_ for 30 min in the live/dead solution (Alexa Fluor-488 Calcein AM and Alexa Fluor-568 Ethidium homodimer) and observed with fluorescent confocal microscopy (laser scanning microscope 880, Zeiss). An objective EC Plan-Neofluar 10×/NA 0.3 was used for this study. The single images were randomly selected from different areas of each sample.

The digital images were processed using FIJI 2.14.0 image-processing software. Finally, an index of live cells was calculated based on the ratio of living cells (green) to the total number of cells (green + red) in each area. The values represent the mean +/− standard deviation of at least three biological replicates.

#### 2.2.10. Cell Morphology

For a proper understanding of the effect of the hydrogel formulations on cell spreading, hydrogels with and without Gel-TA were prepared as described above. The same concentration of polymers (2.5% *w*/*v* HA-TA plus or minus 0.05% *w*/*v* Gel-TA) was used. A single cell-laden hydrogel (10 million cells/mL) was placed in triplicate in a 12-well plate. Morphology was assessed on day 7 as the cultured cell-laden hydrogels were fixed with 4% formaldehyde, permeabilized with 0.5% *v*/*v* Triton X 100, and stained with Alexa Fluor-546 Phalloidin, as well as Alexa Fluor-405 DAPI stains for F-actin and the nuclei. The same confocal laser scanning microscope with objective LD Plan-Neofluar 20×/0.40 Korr M27 was used for this study.

Single images were randomly selected from different areas of each sample and processed using FIJI software. Cell shape was captured based on the presence of actin filaments (orange) and a nucleus (blue) in each area. The images were also processed using FIJI 2.14.0 software. 

#### 2.2.11. Immunohistochemistry Staining (IHC)

All samples were washed twice with PBS and fixed in 4% formaldehyde for 30 min at room temperature, then washed twice with PBS and 0.2% Tween 20 for 5 min. The samples were placed in 30% sucrose for 6 h to allow the hydrogel samples to sink. All samples embedded in cryo-matrix were snap-frozen with the use of dry ice/isopentane. Cryosections of 10 µm were cut using a cryotome at −25 °C (Thermo Shandon FSE, Thermo Fisher Scientific, Waltham, MA, USA).

For the immunohistochemistry, cryosections were incubated with 0.1% pepsin and HCL at 37 °C for 30 min, washed in 0.1% PBS and Tween (PBST), then, blocked in 0.3% H_2_O_2_ for 10 min and washed and blocked in 5% bovine serum albumin (BSA) for 30 min at room temperature. The slides were then incubated overnight at 4 °C with a rabbit polyclonal primary antibody against COL II (Abcam, Cambridge, UK). Subsequently, the sections were incubated with a polyclonal goat anti-rabbit HRP-conjugated secondary antibody (Dako, Glostrup, Denmark) followed by development with a DAB substrate kit (Abcam).

Counterstaining was performed with haematoxylin. Non-immune controls underwent the same procedure without primary antibody incubation. The immunohistochemistry-stained slides were scanned with a NanoZoomer 2.0-RS slide scanner (Hamamatsu, Sendai City, Japan) to obtain images of the stained cells in the cell-laden hydrogels.

#### 2.2.12. Statistical Analysis

A two-tailed, paired-sample *t*-test was conducted using OriginPro 2023 to compare the mean values of paired groups. The analysis was performed at a 95% confidence level, with statistical significance defined as *p* < 0.05, high significance as *p* < 0.01, and extreme significance as *p* < 0.001.

## 3. Results and Discussion

### 3.1. Hydrogel Formation and Gelation Time

Previous research has demonstrated the effectiveness of enzymatic crosslinking in tyramine-modified polymers. Jin et al. [18] highlighted that the gelation time of dextran-tyramine hydrogels can be controlled by varying the concentrations of horseradish peroxidase (HRP) and hydrogen peroxide (H_2_O_2_), emphasizing the critical role of enzymatic crosslinker concentration in forming stable networks. Similarly, Darr and Calabro [24] reported that the gelation kinetics of tyramine-functionalized hyaluronic acid hydrogels are influenced by the degree of substitution (DS) and enzyme levels, with higher crosslinking densities resulting in faster gelation. Further, Jin et al. [17] demonstrated that the interplay between polymer composition and enzymatic activity significantly affects gelation times in injectable dextran-hyaluronic acid hydrogels. These studies provide a valuable framework for understanding how factors such as molecular weight, crosslinker concentration, and functional group availability work together to govern gelation time and mechanical properties.

**Figure 1:**
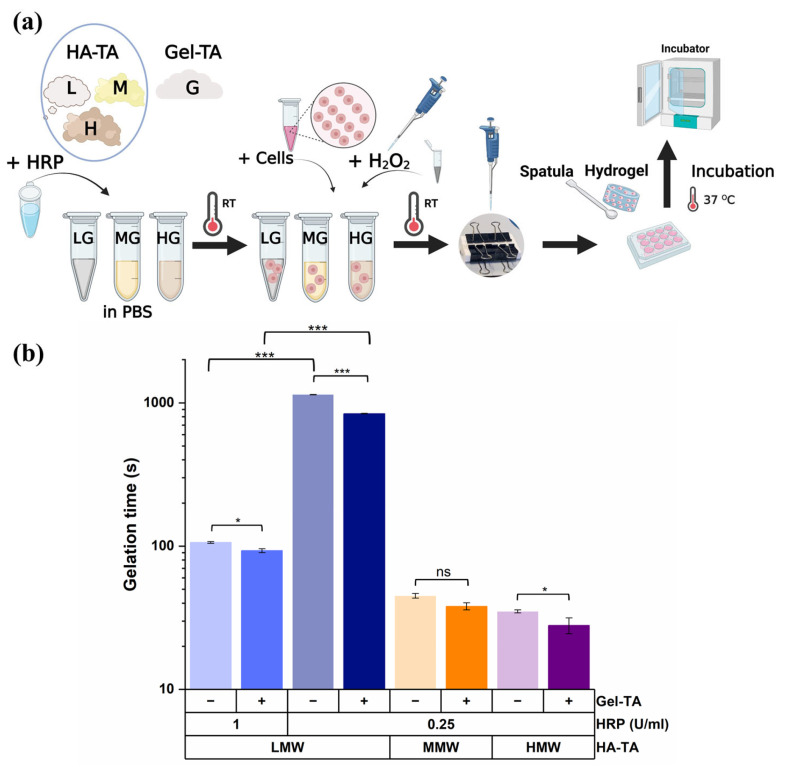
(**a**) Schematic workflow of cell encapsulation and hydrogel formation for cell culture. LG represents LMW HA-TA/Gel-TA, MG represents LMW HA-TA/Gel-TA, and HG represents HMW HA-TA/Gel-TA; (**b**) time of gelation for all formed hydrogels consisting of LMW, MMW, and HMW HA-TA having a DS of 10, 10, and 6.5%, respectively, at polymer concentrations of 2.5% *w*/*v* with and without 0.05% *w*/*v* Gel-TA (DS 16.5%). These gels were produced with 0.25 or 1 U/mL HRP and 0.00474% H_2_O_2_. The asterisks (* and ***) are used to indicate different levels of statistical significance based on the following *p*-values: ns: *p* > 0.05 (not significant), *: *p* < 0.05 (significant), and ***: *p* < 0.001 (extremely significant).

Building on this foundation, in our study, a more detailed description of hydrogel formation was considered. Hydrogels were prepared by dissolving the tyramine-functionalized polymers (LMW HA-TA DS 10%, MMW HA-TA DS 10%, and HMW HA-TA DS 6.5% with and without Gel-TA DS 16.5%) and HRP in PBS (with the presence of cells where needed). Afterward, H_2_O_2_ was added as the oxidizing agent. Crosslinking of tyramine was established when H_2_O_2_ was added and the time of gelation was measured by the vial-tilting method (Figure 1). Despite the lower DS of HMW HA-TA, we added the same amount of H_2_O_2_ to obtain similar amounts of TA:TA crosslinks. Neither MW nor Gel-TA addition influenced the size of the hydrogels (Supplementary Materials: Figure S3). The experimental procedure is shown in Figure 1a. The use of 0.25 U/mL HRP for all conditions and an additional concentration of 1 U/mL HRP for LMW HA-TA and LMW HA-TA/Gel-TA enabled us to have a suitable working time (Figure 1b).

Figure 1b shows a significant difference in gelation time between LMW HA-TA with and without Gel-TA at 1 U/mL HRP and HMW HA-TA with and without Gel-TA (*p* < 0.05, *). In addition, an extremely significant difference was found between LMW HA-TA with and without Gel-TA at 0.25 U/mL HRP (*p* < 0.001, ***), as well as between HRP concentrations (0.25 and 1 U/mL) in LMW HA-TA and LMW HA-TA/Gel-TA. The gelation time decreased as the HRP concentration increased from 0.25 to 1.0 U/mL, likely due to enhanced catalytic activity in the enzyme, which accelerated the crosslinking process. This trend is consistent with findings by Zhong et al. [25], who demonstrate that higher HRP concentrations promote faster gelation by increasing the rate of enzymatic reactions involved in forming the hydrogel network. Generally, the observed trend shows that gelation time decreases with an increase in molecular weight (MW) and/or the addition of Gel-TA in tyramine-modified polymers. Higher MW polymers form denser networks due to greater chain entanglement and functional group availability while Gel-TA adds more tyramine and tyrosine residues, enhancing enzymatic crosslinking. For further understanding, you may refer to studies by Mayol et al. [26] and Hasturk et al. [27], which explore the impact of molecular weight and additional material on gelation kinetics in tyramine-based hydrogels.

### 3.2. Mechanical Properties

**Figure 2:**
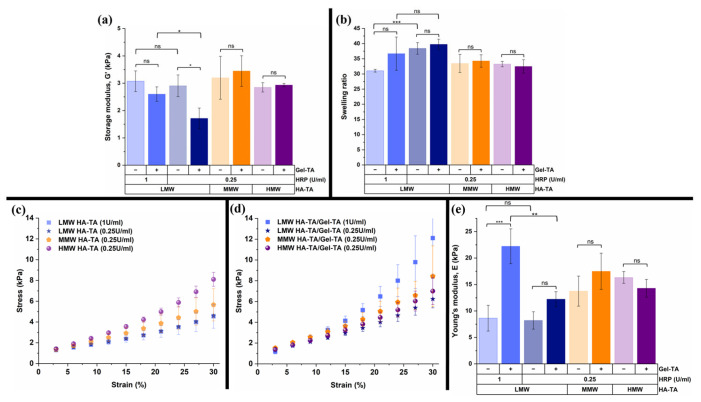
(**a**) Storage modulus of all hydrogel compositions with and without Gel-TA hydrogel crosslinked with 0.25 and 1 U/mL HRP. (**b**) Swelling ratio of all hydrogel compositions, including 2.5% *w*/*v* LMW HA-TA with and without Gel-TA hydrogel crosslinked with 0.25 and 1 U/mL HRP. (**c**) Stress and strain curve of HA-TA and (**d**) HA-TA/Gel-TA and the (**e**) corresponding Young’s modulus values. The cylindrical hydrogel samples had an increase in the molecular weight of HA-TA with and without Gel-TA and equal crosslinking, i.e., the amount of H_2_O_2_. These gels were produced with 0.25 and 1 U/mL HRP. At least three hydrogel samples per concentration were tested. The asterisks (*, **, and ***) are used to indicate different levels of statistical significance based on the following *p*-values: ns: *p* > 0.05 (not significant), *: *p* < 0.05 (significant), **: *p* < 0.01 (highly significant), and ***: *p* < 0.001 (extremely significant).

The storage modulus, Young’s modulus, and equilibrium swelling ratio of the cylindrical hydrogels are depicted in Figure 2. In Figure 2a, it was observed that the molecular weight of HA did not affect the storage modulus of the hydrogels. However, research shows that an increase in the MW of HA reinforces the 3D network of the polymer, leading to higher viscoelastic properties such as the storage modulus [5]. There was a significant difference between HA-TA/Gel-TA with 0.25 U/mL HRP and 1 U/mL HRP (*p* < 0.05, *) but no significant difference was observed between HA-TA with 0.25 U/mL HRP and 1 U/mL HRP concentrations above *p* > 0.05, suggesting Gel-TA concentration may have influenced the outcome. Gel-TA may have introduced more functional groups that enable enhanced crosslinking with HA-TA when using a higher HRP concentration (1 U/mL) compared to HA-TA alone. This effect is more pronounced because Gel-TA provided additional tyramine and tyrosine residues that may also have participated in HRP/H₂O₂-mediated crosslinking, further increasing the degree of network formation [28,29,30]. The HA-TA and HA-TA/Gel-TA hydrogels have storage moduli between 1.8 and 3.4 kPa; these values are lower than in the 2.2% *w*/*v* HMW HA-TA hydrogels (~5 kPa) reported by Banigo et al. [31] and occurred partly due to the lower HRP and H_2_O_2_ concentrations aimed at 50% crosslinking of the tyramine residues [32].

The Young’s modulus under low strain (Figure 2e), obtained from the stress/strain curve in Figure 2c,d, was found to increase with the increase in the MW of HA and addition of Gel-TA, as expected based on previous research [5], except for HMW HA-TA/Gel-TA. This discrepancy might be caused by the high viscosity of the hydrogel precursor, which could hinder efficient crosslinking and limit the formation of a fully developed hydrogel network. Additionally, the Young’s modulus increased as the HRP concentration increased in LMW HA-TA and HA-TA/Gel-TA hydrogels. Figure 2e shows an extremely significant difference between LMW HA-TA and LMW HA-TA/Gel-TA (1 U/mL HRP) (*p* < 0.05, ***) and a highly significant difference between LMW HA-TA/Gel-TA with 0.25 U/mL HRP and 1 U/mL HRP (*p* < 0.01, **). Other conditions had no significant difference (ns) based on their statistical plotted data. Our hydrogels have Young’s moduli <25 kPa (Figure 2e) and can store energy elastically. The 2.5% *w*/*v* HMW HA-TA and HMW HA-TA/Gel-TA possess a Young’s moduli of 16.3 kPa and 14.3 kPa. They are comparable to the Young’s modulus of 2.2% *w*/*v* HMW HA-TA hydrogels (~13.3 kPa) used for coaxial bioprinting [31].

Next, we studied the swelling properties of the hydrogels. According to Snetkov et al. [5], MW increase leads to more hydrogen bonds in single HA molecules, which results in the greater entanglement of HA chains, and with more crosslinking points, the swelling ratio also decreases. The swelling ratio indicates a decrease in network density due to degradation [33]. In this study, the MW of HA influenced the swelling ratio, with LMW HA-TA having a higher swelling ratio compared to MMW and HMW HA-TA independent of Gel-TA addition as predicted, based on Snetkov et al. [5]. Next to that, the HRP concentration influenced the swelling ratio of both LMW HA-TA and LMW HA-TA/Gel-TA hydrogels. Increasing the HRP concentration led to a highly significant decrease in swelling in LMW HA-TA (*p* < 0.001, ***), as the swelling ratio decreased from 38 to 31. However, when including Gel-TA, the decrease in the swelling ratio caused by the HRP concentration was not significant (ns), with a swelling ratio decrease from 39 to 36. Furthermore, there was no significant difference between any other condition with or without Gel-TA (Figure 2b), indicating the swelling ratio significantly changed in LMW HA-TA due to the HRP concentration based on a two-sample *t*-test.

**Figure 3:**
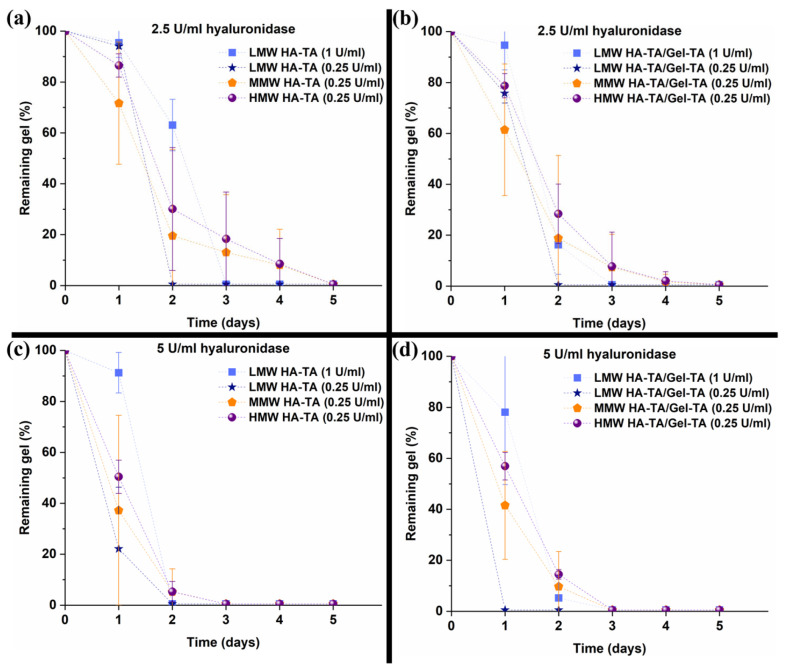
(**a**) The degradation of hydrogels by hyaluronidase depending on the MW of the HA-TA and addition of Gel-TA in (**a**,**b**) 2.5 U/mL hyaluronidase and (**c**,**d**) 5.0 U/mL hyaluronidase.

HA, as an important part of the ECM in cartilage tissue, can be degraded by hyaluronidase [34,35]. As shown in Figure 3, increasing the MW of HA-TA prolonged the degradation time in 2.5 and 5 U/mL hyaluronidase. The LMW hydrogels with 1 U/mL HRP were less susceptible to degradation than those with 0.25 U/mL HRP, likely due to more efficient crosslinking. The addition of Gel-TA did not significantly impact the degradation rate, as shown in Figure 3.

### 3.3. Cell Viability and Morphology of Chondrocytes in the Different Hydrogels

**Figure 4:**
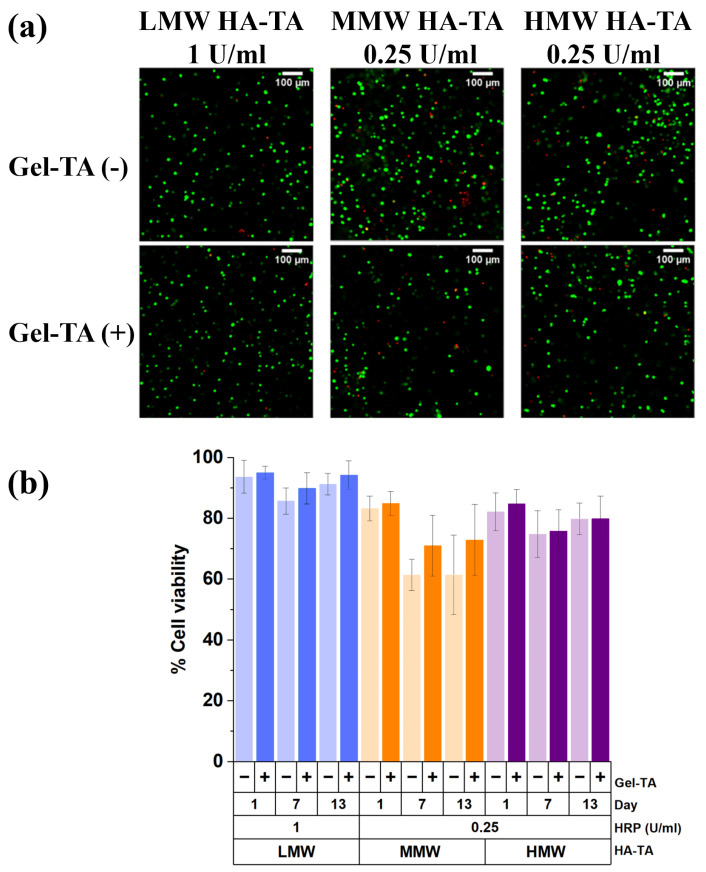
Impact of MW in HA-TA with and without Gel-TA on cell viability. (**a**) Images of crosslinked BPC-laden constructs on day 1, stained for live (green) and dead (red) cells. (**b**) Corresponding cell viability plots of the crosslinked cell-laden constructs (BPCs) with and without Gel-TA, including 2.5% *w*/*v* LMW HA-TA with and without Gel-TA hydrogel with 1 U/mL HRP on days 1, 7, and 13.

The use of low HRP and H_2_O_2_ concentrations in these hydrogels produced biocompatible cell-laden constructs confirming previous results by Wang et al. [36]. The bovine primary chondrocytes were homogeneously distributed (Figure 4a). Cell-laden hydrogels made of LMW polymers had high cell viability which remained remarkably stable over time with a viability above 90% at day 13. The viability in hydrogels made of HMW polymers remained stable over time with a lower viability of 80% (Figure 4b).

Zhou et al. [37] demonstrated good fibroblast viability in macroporous hydrogels containing gelatin with live/dead staining showing consistent viability across varying MgMP concentrations over 1, 3, and 5 days. In contrast, our study focused on chondrocytes within hydrogels containing Gel-TA. While cell viability remained high overall, MMW hydrogels showed reduced viability (<80%) at days 7 and 13, which improved with the addition of Gel-TA. However, in LMW and HMW hydrogels, the addition of Gel-TA had no significant impact on viability. This comparison highlights the distinct responses of cell types and hydrogel compositions, underscoring the context-dependent effect of gelatin incorporation.

### 3.4. Cell Morphology and Histology

**Figure 5:**
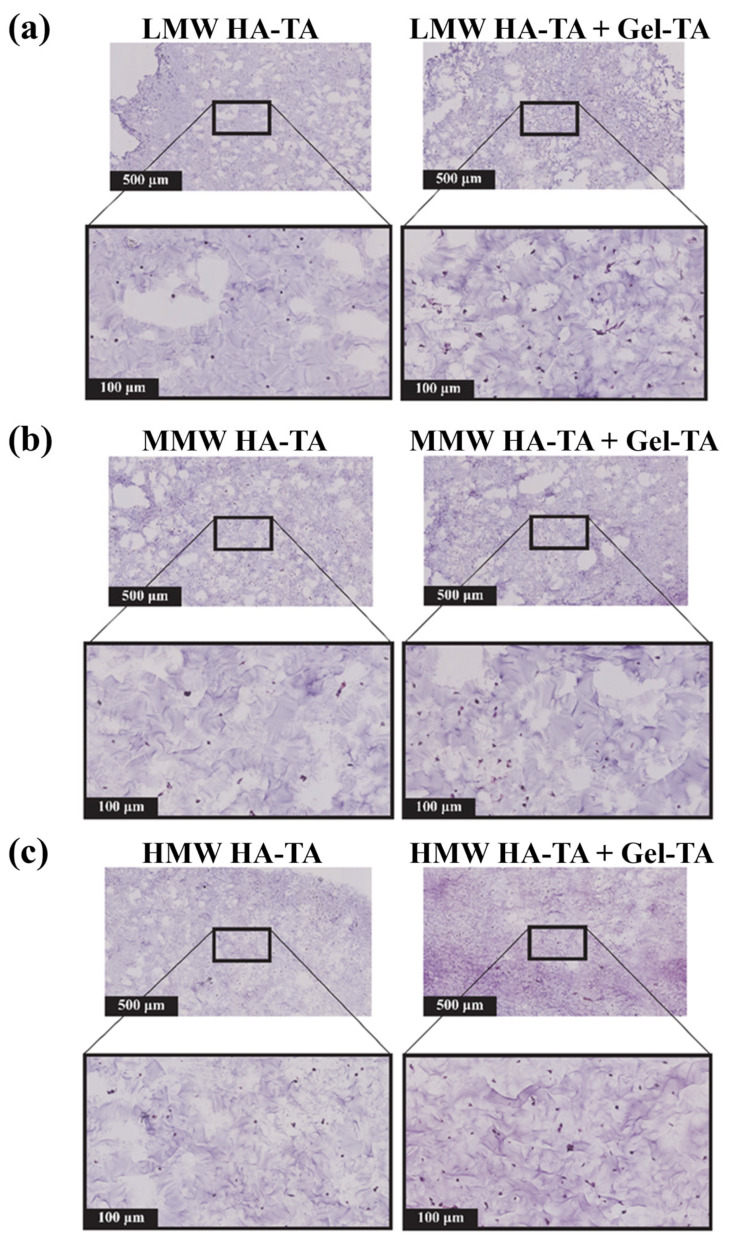
Histology of BPCs encapsulated in 2.5% *w*/*v* hydrogels after culturing for 13 days in a chondrogenic medium. The representative histological sections of hydrogels stained with H&E are shown: (**a**) LMW HA-TA and LMW HA-TA/GEL-TA, (**b**) MMW HA-TA and MMW HA-TA/GEL-TA, and (**c**) HMW HA-TA and HMW HA-TA/GEL-TA. The top panel in each condition shows 5× magnification images (scale bars represent 500 µm) whereas the right panel shows 20× (scale bars represent 100 µm) magnification.

The morphology, attachment, and proliferation of cells were studied, revealing the significant influence of Gel-TA on cellular behavior across hydrogel formulations. Cells in HA-TA/Gel-TA hydrogels began to spread (as shown in Supplementary Materials: Figure S4), attach, and proliferate (as shown in Figure 5), while cells in HA-TA-only hydrogels remained round. Due to rapid degradation during cell culturing, LMW HA-TA/Gel-TA hydrogels were excluded from further analysis. These observations align with previous findings by Hendriks et al., who highlight the role of Gel-TA in promoting cell attachment and proliferation in tyramine-modified hydrogels [3].

Gel-TA enhanced cell viability, spreading, and cartilage matrix formation. In MMW HA-TA/Gel-TA formulations, cell survival and spreading improved compared to MMW HA-TA alone, suggesting Gel-TA provides a more supportive microenvironment. Similarly, Gel-TA promoted better cell spreading in MMW and HMW HA-TA/Gel-TA hydrogels, whereas no spreading was observed in LMW HA-TA/Gel-TA due to rapid degradation. Gel-TA likely improves cell adhesion by offering additional binding sites for integrins and other receptors critical for tissue formation.

**Figure 6:**
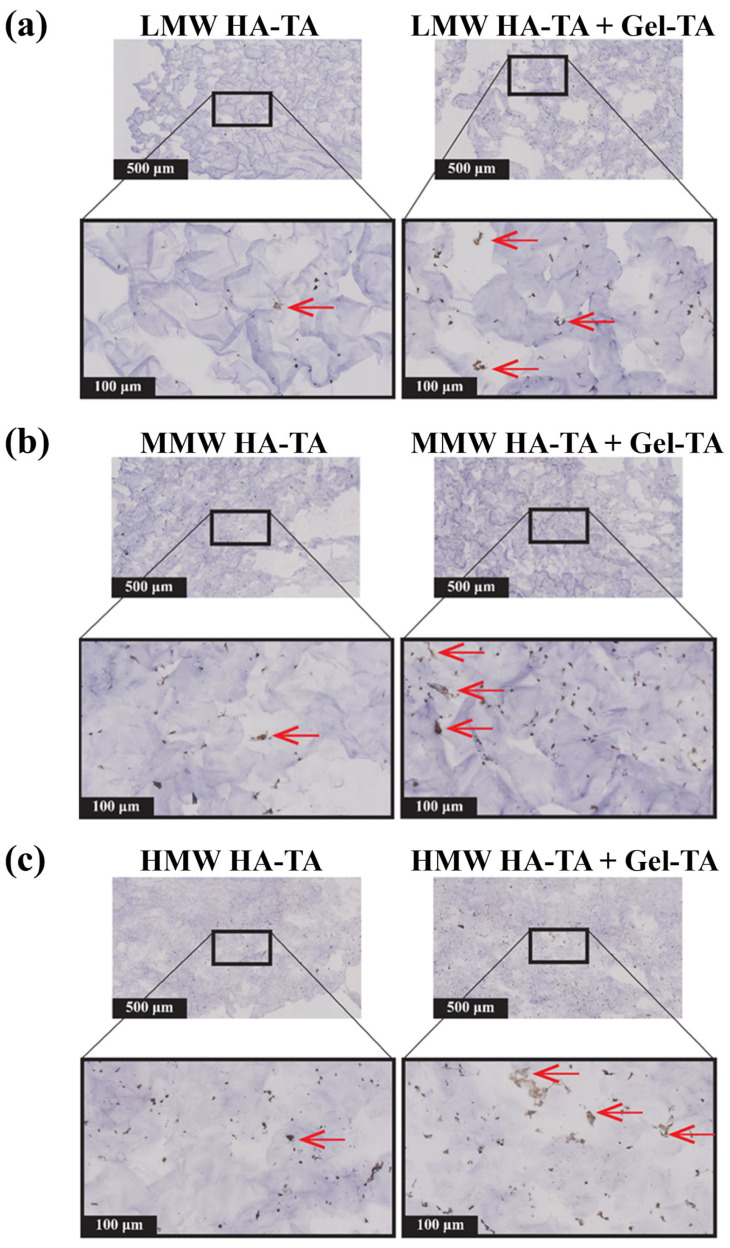
Immunohistochemistry staining of collagen type II is dependent on the MW of HA-TA and the presence of Gel-TA. Over 13 days, 2.5% *w*/*v* hydrogels encapsulated with bovine primary chondrocytes were cultured in a chondrogenic medium. Representative immunohistochemical staining of the hydrogels for Col II. positive protein expression were stained in dark brown for all conditions. (**a**) LMW HA-TA and LMW HA-TA/GEL-TA, (**b**) MMW HA-TA and MMW HA-TA/GEL-TA, and (**c**) HMW HA-TA and HMW HA-TA/GEL-TA. The red arrows point to the regions with collagen type II deposition. The top panel in each condition shows 5× magnification pictures (scale bars represent 500 µm) whereas the bottom panel shows 20× (scale bars represent 100 µm) magnification.

Histological and immunohistochemical analyses further demonstrated Gel-TA’s role in enhancing cartilage matrix deposition, particularly in MMW and HMW HA-TA/Gel-TA hydrogels, supporting the synthesis of extracellular matrix proteins like collagen (Figure 6 and Figure S5). Increased stiffness in these hydrogels appeared to facilitate matrix formation, while softer LMW HA-TA/Gel-TA hydrogels, although supportive of higher cell viability, exhibited reduced matrix deposition.

After 13 days, immunohistochemical staining confirmed the formation of collagen type II in Gel-TA-enhanced hydrogels, with the highest deposition observed in MMW and HMW HA-TA/Gel-TA formulations. These results align with previous studies, where HMW HA mitigated cartilage degeneration and chondrocyte loss [38]. Lower collagen type II deposition in LMW HA-TA hydrogels reflects findings by Fu et al., where similar formulations displayed limited matrix formation [9].

Overall, the incorporation of Gel-TA in HA-TA hydrogels supports cell proliferation, spreading, and matrix synthesis, highlighting its potential utility in cartilage tissue engineering.

## 4. Conclusions

Our study demonstrates the promising potential of enzymatically crosslinkable low, medium, and high molecular weight hyaluronic acid-tyramine (LMW, MMW, and HMW HA-TA) hydrogels with and without gelatin-tyramine (Gel-TA) for cartilage tissue engineering applications. The incorporation of Gel-TA into HA-TA hydrogels significantly enhanced the biological properties, promoting better cell attachment and proliferation over time. Notably, the presence of Gel-TA in MMW and HMW HA-TA hydrogels resulted in the highest levels of cartilage matrix formation.

Our findings suggest that the molecular weight of HA influences cartilage matrix formation, a phenomenon that cannot be solely attributed to the stiffness of the hydrogel. This underscores the importance of optimizing the molecular composition of hydrogel to achieve desired tissue engineering outcomes.

The fine-tuned properties and good cellular behaviour of these hydrogels make them suitable for further exploration in cartilage and other soft tissue engineering studies. Overall, this study highlights the potential of these HA-TA hydrogels with the addition of Gel-TA for cartilage tissue engineering.

## Data Availability

The original contributions presented in the study are included in the article/Supplementary Materials, further inquiries can be directed to the corresponding author.

## Supplementary Materials

The following supporting information can be downloaded at: https://www.mdpi.com/article/10.3390/polym16233410/s1, Protocol 1a. Synthesis of low and medium molecular weight hyaluronic acid-tyramine; Protocol 1b. Synthesis of high molecular weight hyaluronic acid-tyramine; Table S1. Some properties of the different molecular weights of hyaluronic acid; Figure S1: Chemical structure of Gel-TA and HA-TA conjugates of different molecular weights and hydrogel formation in the presence of HRP and H_2_O_2_ as enzymatic crosslinking agents; Figure S2: Sketch of the PTFE mold used for hydrogel formation; Figure S3: Formed hydrogels of hyaluronic acid with low, medium, and high molecular weights and gelatin functionalized with tyramine having different degrees of substitution (DS); Figure S4: Morphology of BPCs in cell-laden hydrogels; and Figure S5: Immunohistochemistry staining of collagen type II for 2.5% *w*/*v* hydrogels encapsulated with chondrocytes after culturing for 13 days in chondrogenic medium. References [39,40,41,42,43,44,45,46,47,48,49] are cited in the Supplementary Materials
