# Supplementary material for: Synthesis and Engineering of Hyaluronic Acid-Gelatin Hydrogels with Improved Cellular Attachment and Growth
Source: Polymers (Basel). 2024 Dec 3;16(23):3410. doi: 10.3390/polym16233410 (PMC11644312; doi:10.3390/polym16233410)
Supplement: Supplementary file 1 [file polymers-16-03410-s001.zip › polymers-3291339-supplementary.pdf]

Supplementary information

Protocols

1a. Synthesis of low and medium molecular weight hyaluronic acid-tyramine

5.0 g of sodium hyaluronate (27 kDa or 298 kDa, 12.5 mmol r.u) was dissolved in 500 mL Milli-Q water in a 1 L round bottom flask equipped with a stirrer bar. While stirring at room temperature, 4-(4,6-dimethoxy-1,3,5- triazin-2-yl)-4-methylmorpholinium chloride (DMTMM, 3.46 g, 12.5 mmol, 1 eq) and tyramine hydrochloride (TA·HCl, 2.17 g, 12.5 mmol, 1 eq) were added subsequently.

The addition of DMTMM and TA·HCl was repeated after 24 and 48h. After 72h, 40ml of saturated NaCl was added to the reaction mixture. Afterwards the reaction mixture was precipitated in 2.5 L cold ethanol. The crude product was thoroughly washed with 250 ml ethanol (x4) and 100 ml Diethyl ether using a Porr 4 filter followed by drying under vacuum for 3 days. The crude product was dissolved in 75 mL Milli-Q water and dialysed against Milli-Q water for 3 days (MWCO 1000 Da). Filter sterilization and lyophilization yielded the product as a white foam (5.10 g, 12.4 mmol, 99 % yield, DS 10 %). ^{1}H-NMR (400 MHz, D_2O): δ(ppm) = 1.98 (acetyl-CH_3, s, 3H); 2.75 (2-CH_2, s, 2H); 2.90 (1-CH_2, s, 2H); 3.2-4.2 (saccharide ring, m, 10H); 4.34 (s, 1H); 4.43 (d, 1H); 6.84 (Ar m-CH, d, 2H); 7.16 (Ar o-CH, d, 2H).

1b. Synthesis of high molecular weight hyaluronic acid-tyramine

5.0 g of sodium hyaluronate (2-2.2 MDa, 12.5 mmol r.u) was dissolved in 1 L Milli-Q water in a 2 L round bottom flask equipped with a stirrer bar. While stirring at room temperature, 4-(4,6-dimethoxy-1,3,5- triazin-2-yl)-4-methylmorpholinium chloride (DMTMM, 3.46 g, 12.5 mmol, 1 eq) and tyramine hydrochloride (TA·HCl, 2.17 g, 12.5 mmol, 1 eq) were added subsequently. After 24 hours, 200 mL saturated NaCl solution was added slowly to the reaction mixture. Afterwards the reaction mixture was precipitated in 3 L of cold ethanol. The crude product was thoroughly washed with 250 ml ethanol (x4) and 100 ml Diethyl ether using Porr 4 filter followed by drying under vacuum for 3 days. The crude product was dissolved in 75 mL Milli-Q water and dialysed against Milli-Q water for 5 days (MWCO 6-8 kDa) and lyophilized to yield a white foam (5.10 g, 12.4mmol, 99 % yield, DS 6.5 %).

^{1}H-NMR (400 MHz, D_2O): δ(ppm) = 1.98 (acetyl-CH_3, s, 3H); 2.75 (2-CH_2, s, 2H); 2.90 (1-CH_2, s, 2H); 3.2-4.2 (saccharide ring, m, 10H); 4.34 (s, 1H); 4.43 (d, 1H); 6.84 (Ar m-CH, d, 2H); 7.16 (Ar o-CH, d, 2H).

The degree of substitution (DS) was calculated based on the integral of the methyl group at 1.98 ppm as compared to the integral of the tyramine signals at 6.80-6.87 and 7.10-7.21 ppm. The DS of hyaluronic acid is given as the percentage of COOH groups modified in hyaluronic acid (i.e. per disaccharide).

Supplementary Tables

Table S1. Some properties of the different molecular weights of hyaluronic acid.

Good (+), Better (++), Lacks (-)

	Hyaluronic acid			References
	Low (<250 kDa)	Medium (250-1000 kDa)	High (>1000 kDa)	
**Anti-inflammatory properties**	-	+	+	39–41
**Cell proliferation and mobility *in vivo***	++	+	+	41,42
**Collagen Type II expression**	+	++	++	43
**Chondroadherin (CHAD) expression**	+	++	++	43
**Chondroprotection**	+	+	+	44–46
**Elevate the tissue integrity**	+	++	++	47
**Effective tissue penetration**	++	+	-	44
**Hypoxia-inducible factor 1α (HIF-1α)**	+	++	++	43
**Viscoelastic properties**	+	++	+++	48
**Space filler and natural immunologic depressant**	+	++	++	49
**Viscosity**	+	++	+++	48
**Joint lubrication and shock absorption**	+	++	++	8

Supplementary Figures

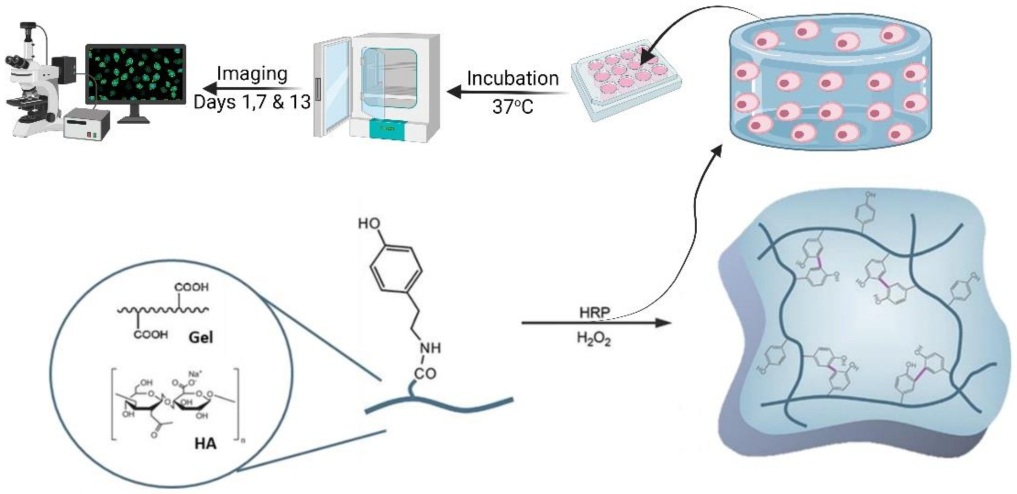

Figure S1: Chemical structure of Gel-TA and HA-TA conjugates having different molecular weights, and hydrogel formation in the presence of HRP and H_2O_2 as the enzymatic crosslinking agents [42]{www.biorender.com}.

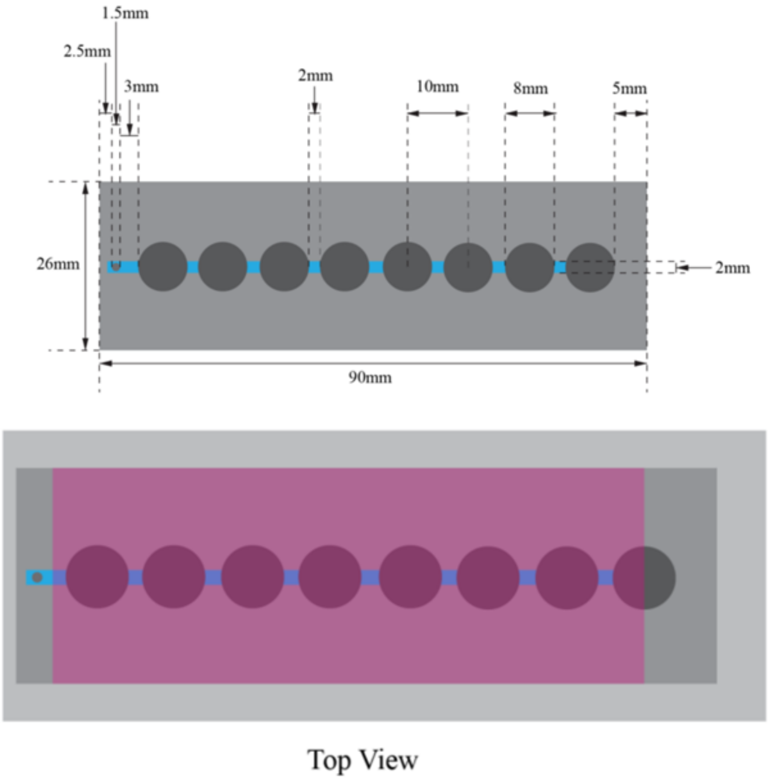

Figure S2: The sketch of the PTFE mold used for hydrogel formation.

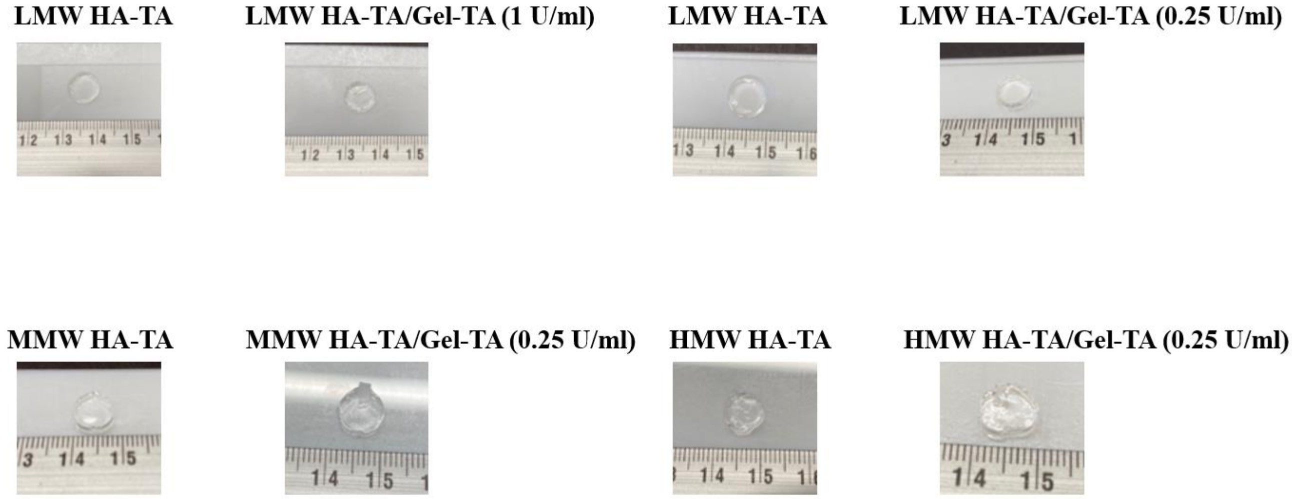

Figure S3: Formed hydrogels of hyaluronic acid with low, medium, or high molecular weights and gelatin functionalized with tyramine having different degrees of substitution (DS). The HRP concentration was 0.25 U/ml for all conditions and additional 1 U/ml HRP for LMW polymers, and the H_2O_2 concentration was 0.00474%.

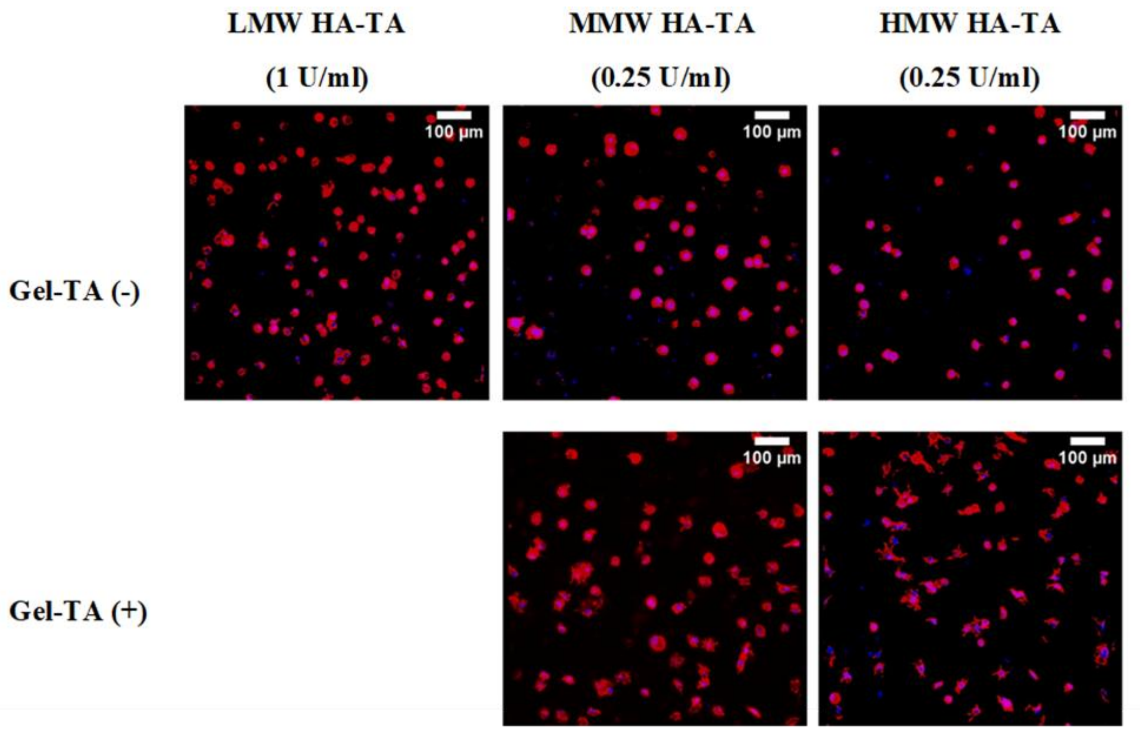

Figure S4: Morphology of BPCs in cell-laden hydrogels. The BPCs were stained for F-actin and nuclei on Day 7, revealing their rounded shape for compositions without Gel-TA and slightly stretched shape for compositions with Gel-TA, respectively.

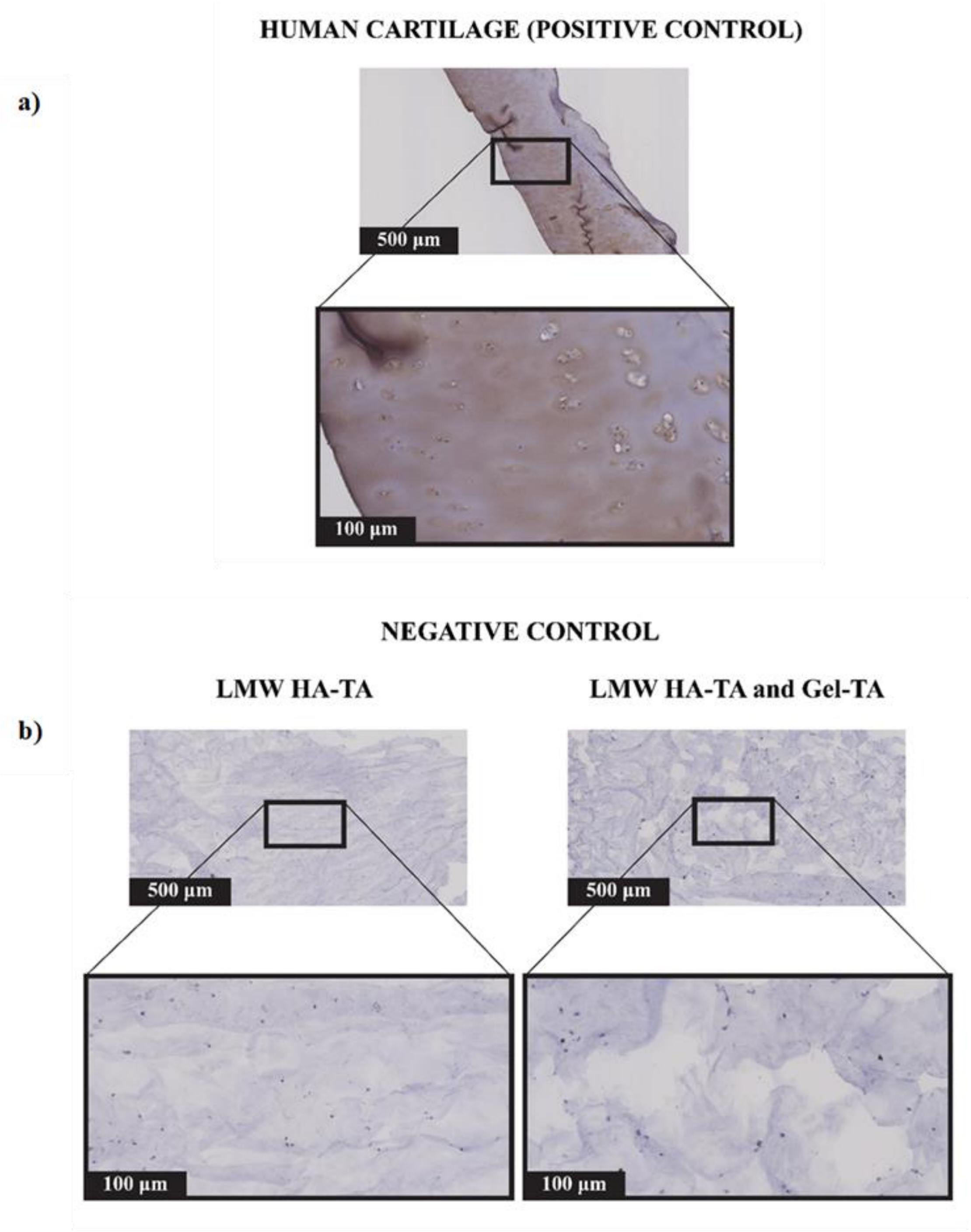
HUMAN CARTILAGE (POSITIVE CONTROL)
a)
500 µm
100 µm
NEGATIVE CONTROL
LMW HA-TA
LMW HA-TA and Gel-TA
b)
500 µm
500 µm
100 µm
100 µm

c)

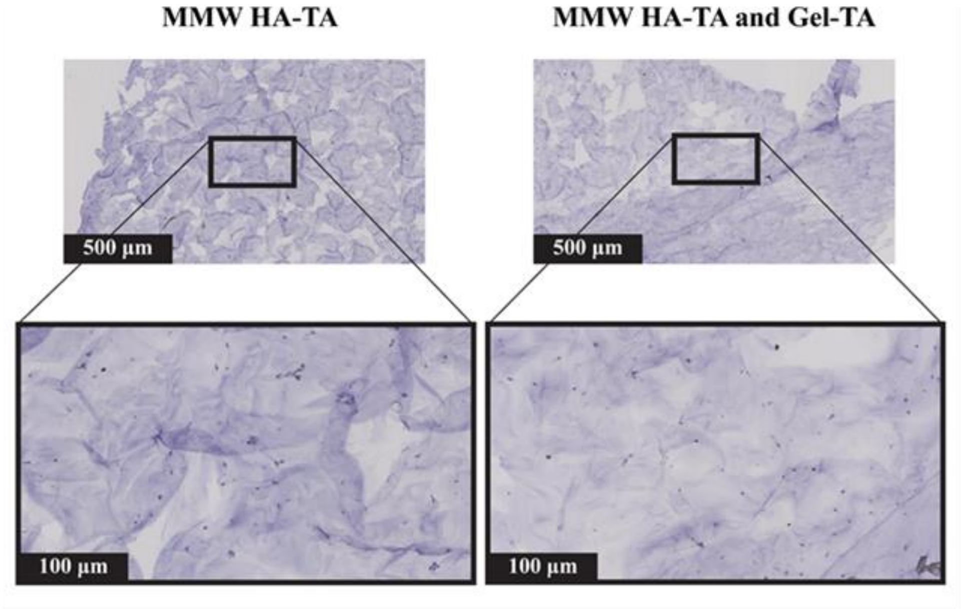

d)

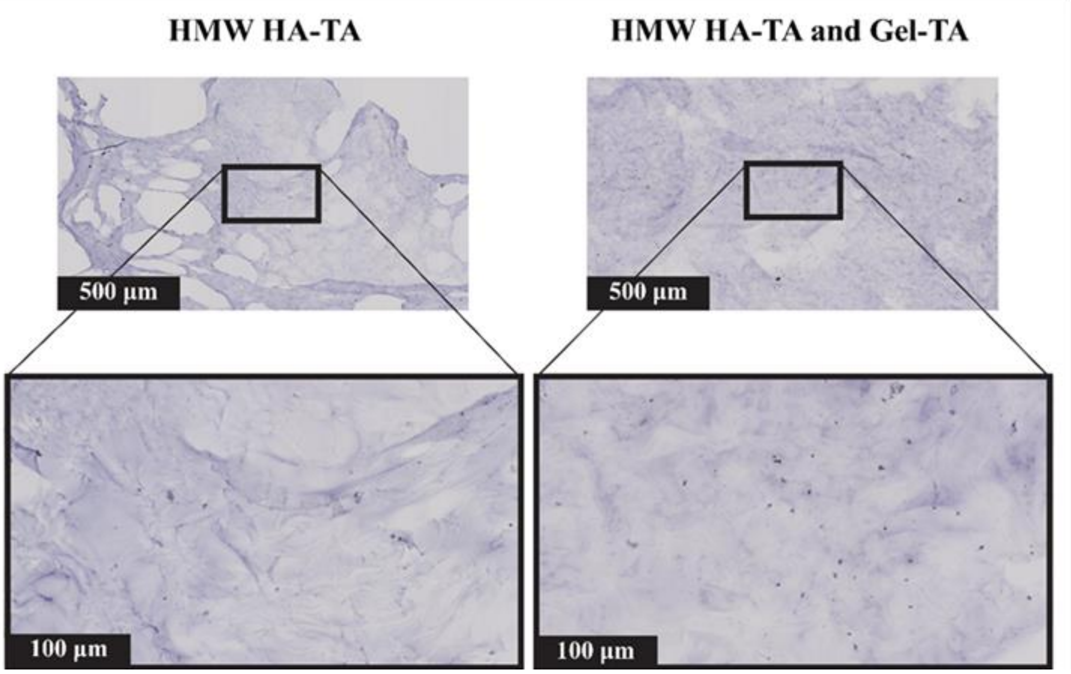

Figure S5: Immunohistochemistry staining of collagen type II for 2.5 %w/v hydrogels encapsulated with chondrocytes after culturing for 13 days in chondrogenic medium. Representative immunohistochemical staining of the control samples (hydrogels) for Coll II. No positive protein staining was observed. a) Human cartilage b) LMW HA-TA and LMW HA-TA/GEL-TA c) MMW HA-TA and MMW HA-TA/GEL-TA d) HMW HA-TA and HMW HA-TA/GEL-TA. The top panel in each condition shows 5× magnification pictures (scale bars represent 500 µm), whereas the right panel shows 20× (scale bars represent 100 µm).
